# Impaired Adaptive Response to Mechanical Overloading in Dystrophic Skeletal Muscle

**DOI:** 10.1371/journal.pone.0035346

**Published:** 2012-04-12

**Authors:** Pierre Joanne, Christophe Hourdé, Julien Ochala, Yvain Caudéran, Fadia Medja, Alban Vignaud, Etienne Mouisel, Wahiba Hadj-Said, Ludovic Arandel, Luis Garcia, Aurélie Goyenvalle, Rémi Mounier, Daria Zibroba, Kei Sakamato, Gillian Butler-Browne, Onnik Agbulut, Arnaud Ferry

**Affiliations:** 1 Université Paris Diderot, Sorbonne Paris Cité, CNRS EAC4413, Unit of Functional and Adaptive Biology, Laboratory of Stress and Pathologies of the Cytoskeleton, Paris, France; 2 Université Pierre et Marie Curie-Paris6, Sorbonne Universités, UMR S794, INSERM U974, CNRS UMR7215, Institut de Myologie, Paris, France; 3 Department of Neuroscience, Uppsala University, Uppsala, Sweden; 4 Université Paris Descartes, Sorbonne Paris Cité, INSERM U1016, CNRS UMR8104, Institut Cochin, Paris, France; 5 MRC Protein Phosphorylation Unit, College of Life Sciences, University of Dundee, Dundee, United Kingdom; 6 Université Paris Descartes, Sorbonne Paris Cité, Paris, France; Paris Institute of Technology for Life, Food and Environmental Sciences, France

## Abstract

Dystrophin contributes to force transmission and has a protein-scaffolding role for a variety of signaling complexes in skeletal muscle. In the present study, we tested the hypothesis that the muscle adaptive response following mechanical overloading (ML) would be decreased in MDX dystrophic muscle lacking dystrophin. We found that the gains in muscle maximal force production and fatigue resistance in response to ML were both reduced in MDX mice as compared to healthy mice. MDX muscle also exhibited decreased cellular and molecular muscle remodeling (hypertrophy and promotion of slower/oxidative fiber type) in response to ML, and altered intracellular signalings involved in muscle growth and maintenance (mTOR, myostatin, follistatin, AMPKα1, REDD1, atrogin-1, Bnip3). Moreover, dystrophin rescue via exon skipping restored the adaptive response to ML. Therefore our results demonstrate that the adaptive response in response to ML is impaired in dystrophic MDX muscle, most likely because of the dystrophin crucial role.

## Introduction

Skeletal muscle exhibits a crucial capacity to adapt to changing use. In response to repeated high-force contractions, i.e., mechanical overloading (ML), both absolute maximal force production and fatigue resistance of the muscle markedly increase. Tension, stretch and deformation generated during ML, together with other activity-related signals (*e.g*. muscle action potential, metabolites…), are sensed and in turn activate different intracellular signaling pathways that converge to induce muscle cellular and molecular adaptations, such as hypertrophy and fiber-type conversion, resulting in muscle performance improvement [1], [2], [3], [4], [5].

The gain in absolute maximal force induced by ML is, at least partly, attributed to muscle hypertrophy, since absolute maximal force is roughly proportional to muscle cross-sectional area. Muscle hypertrophy in response to ML results from increased protein synthesis via activation of the mammalian target of rapamycin (mTOR) signaling pathway [6], [7]. More precisely, it is the mTOR complex-1 (mTORC1) that contains mTOR and the rapamycin-sensitive raptor subunit that promotes protein synthesis via the phosphorylation of the initiation factor-4E binding protein-1 and the S6 kinase (S6K) that in turn phosphorylates at the S240/244 site the ribosomal S6 protein (rS6)[1], [2]. Akt is also activated by ML [6] and it is known that genetic activation of Akt promotes muscle hypertrophy [8], [9]. Recently, we demonstrated that the alpha-1 isoform of the AMP-activated protein kinase (AMPK-α 1) plays an important role in limiting muscle growth during ML, via the reduced activation of mTOR signaling [10]. In addition, it has been shown that myostatin is down-regulated in response to ML [11]. Since myostatin deactivates mTOR [12], [13], [14], these results suggest that together with AMPK, myostatin also limits muscle hypertrophy induced by ML. In addition, the myostatin inhibitor follistatin [15] and the stress response genes REDD1 and REDD2 (regulated in development and DNA damage response)[1], [16] are possibly involved in the control of mTOR signaling in response to ML. Of note, the activation of the mitogen-activated protein kinase pathway (MAPK) may be not sufficient to induce muscle growth [17], [18] and a recent study reported that satellite cells are not necessary for a robust ML-induced hypertrophy [19].

The fast/glycolytic -to slow/oxidative fiber conversion is likely the cellular and molecular adaptation responsible for the increase in fatigue resistance induced by ML. Indeed, it is well established that slow/oxidative fibers expressing type-1 and -2a myosin heavy chain (MHC-1 and MHC-2a) are more fatigue-resistant than fast/glycolytic fibers expressing MHC-2x and MHC-2b [20]. Calcineurin/NFAT signaling is a key player in the promotion of the slow/oxidative fiber phenotype [3], [21]. Pharmacological inhibition or genetic loss of calcineurin blocks the fast/glycolytic -to slow/oxidative fiber-type conversion induced by ML [22], [23], [24]. The peroxisome proliferator-activated receptor γ coactivator-1 (PGC-1), peroxisome proliferator-activated receptor (PPAR) and AMPK signaling pathways have also been proposed to promote the formation of slow/oxidative fibers [3], [5], [21], [25].

Dystrophin, a costameric protein, is known to operate as a physical link between the actin cytoskeleton and laminin in the extracellular matrix, allowing likely the force to be transmitted outside of the cell and vice-versa [26]. This is supported by the findings that in the MDX mice that lacks dystrophin, muscle specific maximal force (force adjusted to muscle size) production is reduced [27] whereas exon skipping-mediated dystrophin rescue improves it [28]. Thus, force transmission with the participation of dystrophin possibly contributes to the sensing of the tension/deformation. Dystrophin may be also required for the membrane localization and function of certain components of signaling pathways (e.g. mechanoreceptor, dihydropyridine receptor, nNOS) acting in muscle growth and maintenance [29], [30], [31], [32], [33]. Several studies support this hypothesis by showing aberrant activation of Akt and mTOR, calcineurin, and MAPK pathways in the MDX mice [34], [35], [36], [37], [38]. Together these results suggest that dystrophin may contribute to the mechanotransduction and other activity-signaling pathways mediating muscle adaptations in response to ML.

The general aim of this study was to determine the adaptive response to ML of skeletal muscle from MDX mice, a murine model of Duchenne muscular dystrophy (DMD) causing severe muscle weakness for yet not fully understood reasons. We studied the effect of ML on MDX dystrophic muscle performance (maximal force production, fatigue resistance) and cellular and molecular remodeling (hypertrophy, fiber-type conversion…). ML was induced by surgical removal of the synergic muscles of the plantaris hindlimb muscle, a well-established rodent model [6], [7], [10], [19], [22], [23], [24], [39], [40], [41], [42], [43]. More specifically, we tested the hypothesis that the muscle adaptive response following ML would be decreased in MDX mice as compared to healthy mice. We also determined whether dystrophin rescue via exon skipping would restore dystrophic muscle adaptations to ML. To our knowledge, this has not yet been studied.

## Results

### Reduced gain in muscle performance: maximal force

**Figure 1:**
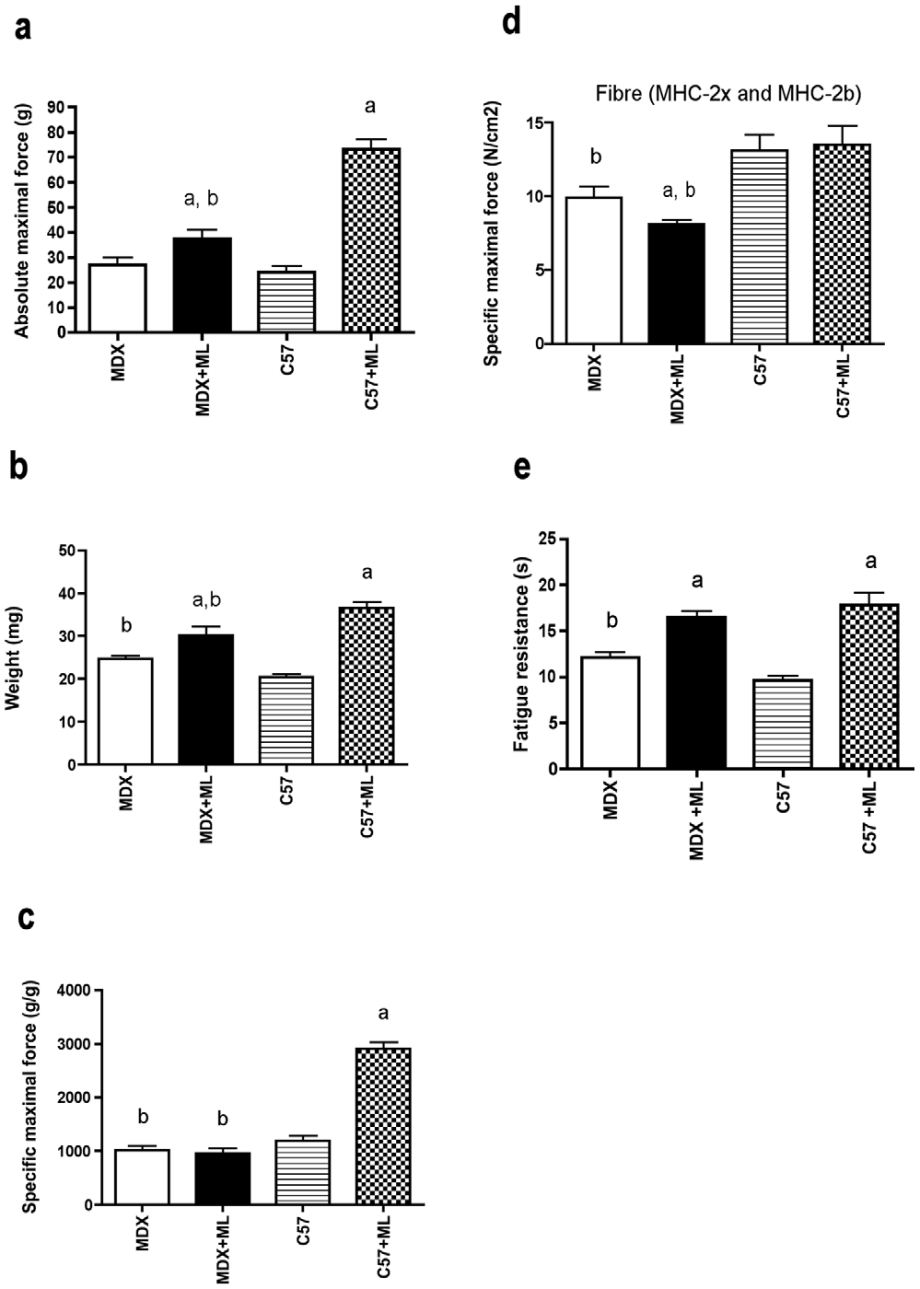
Reduced muscle performance gain and hypertrophy and decrease in contractile **machinery function following 2 month-ML in MDX mice.** Absolute muscle maximal force (a), muscle weight (b), muscle specific maximal force (c), specific maximal force of skinned muscle fibers (d), and muscle fatigue resistance (e) after 2 months of ML. a: significantly different from unoverloaded muscle (p<0.05). b: significantly different from corresponding C57 mice (p<0.05). n = 14–26/group for absolute and specific muscle maximal forces and n = 30/group (at least 7 per animal) for specific maximal force skinned fibers.

*In situ* plantaris muscle force production in response to nerve stimulation showed important changes 2 months after ML. The muscle absolute maximal force in MDX mice with mechanical loading (MDX+ML) and C57+ML mice were markedly increased as compared to MDX mice and C57 mice (Figure 1a). However, muscle absolute maximal force in MDX+ML mice was lower as compared to C57+ML mice, indicating that the increase in muscle absolute maximal force is lower in MDX+ML mice (+40%) as compared to C57+ML mice (+204%) mice. This difference between the 2 strains of mice was first explained by the reduced increase in muscle weight in response to 2-month ML in MDX+ML mice (+24%) as compared to C57+ML mice (+80%) mice (Figure 1b). The fact that in contrast to C57+ML mice, muscle specific maximal force was not increased in MDX+ML mice in response to 2 months of ML (Figure 1c) was also responsible for the smaller increase in muscle absolute maximal force in MDX+ML mice.

To test the possibility that a defect in the contractile machinery (myofilaments) could be directly responsible for the maintained muscle specific maximal force observed in the MDX+ML mice, we also determined the force generation of plantaris skinned muscle fibers in response to maximal activation by calcium 2 months after ML. Data obtained from skinned fibers expressing MHC-2b and MHC-2x were pooled since no difference was observed between these 2 types of fibers. We found that fiber specific maximal force of MDX+ML mice was reduced when compared to MDX mice (−18%) whereas that of C57+ML mice did not change following ML (Figure 1d).

**Figure 2:**
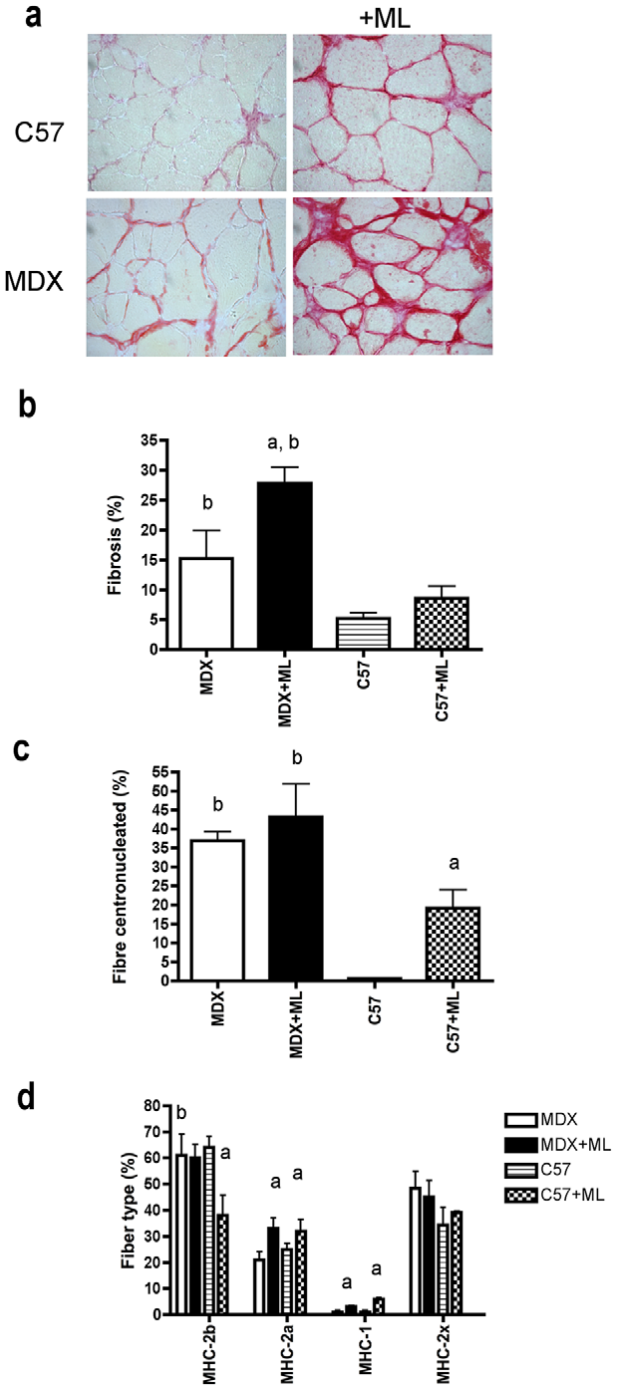
Muscle histology following 2 month-ML in MDX mice. Fibrosis (a and b), percentage of centronucleated muscle fibers (c) and percentage of the different types of muscle fibers (d) after 2 months of ML. Representative images of fibrosis (red) on Sirius red staining cross-section (a) shown increased fibrosis in MDX and MDX+ML mice. Of note, since muscle fiber can express more than one MHC, the total percentage of the different muscle fiber types added up to well over 100%. n = 3–4/group for fibrosis and n = 5–8 for percentage of fibers. a: significantly different from unoverloaded muscle (p<0.05). b: significantly different from corresponding C57 mice (p<0.05).

We also observed an increased fibrosis (decreased concentration of contractile material) in the MDX mice in response to 2 month-ML as shown with Sirius red staining (Figures 2a and 2b), which may partly explain the deficit in muscle specific maximal force observed in MDX+ML. Regenerating fibers were recently reported following ML [19], [54] and are thought to produce less specific maximal force than mature fibers. Therefore, we checked the possibility that ML induced a greater increase in the number of regenerating fibers in MDX+ML mice as compared to C57+ML mice. These regenerating fibers would consequently have a greater contribution to force production in the MDX+ML mice. Nuclei and laminin were stained to count the number of regenerating/regenerated fibers that contain one or more centronuclei 2 months after ML. However, in contrast to C57+ML mice, we observed that there was no increase in the percentage of centronucleated fibers in response to ML in MDX+ML (Figure 2c). No notable expression of neonatal or embryonic MHC was found in both strains 2 months after ML (data not shown), indicating that these centronucleated fibers were regenerated. Since the fast muscle fibers expressing MHC-2b could produce a higher specific maximal force, we tested the possibility that ML reduced more efficiently the percentage of fast fibers in MDX+ML mice as compared to C57+ML mice. However, in contrast to C57+ML mice, immunostaining for MHC isoform revealed that the percentage of fibers expressing MHC-2b did not decrease in MDX+ML mice in response to 2 months ML (Figure 2d). It should ne noted that a substantial number of these fibers co-expressed MHC-2x. Unfortunately, it was not possible to distinguish pure and hybrid fibers expressing MHC-2b.

### Reduced gain in muscle performance: fatigue resistance

Together with the increase in absolute maximal force production, muscles from both MDX+ML mice and C57+ML mice improved their fatigue resistance in response to 2-month ML (Figure 1e). However, the improvement in muscle fatigue resistance was less marked in MDX+ML mice (+36%) as compared to C57+ML mice (+85%). The fact that in contrast to C57+ML mice the percentage of fibers expressing MHC-2b (less fatigue resistant fibers) did not decrease in MDX+ML mice (Figure 2d) might be responsible for this difference. Since the calcineurin/NFAT, PGC-1, and PPAR signaling pathways promote the formation of slow/oxidative/fatigue-resistant fibers [3], we further analysed these pathways 7 days after ML [41] to test the possibility that they are less activated in MDX+ML mice. We found that the transcript levels of NFATc1a, PGC1α1a and PPARß were globally reduced by ML in both mice strains (Figure S1a–c).

### Fiber atrophy and altered catabolic processes

**Figure 3:**
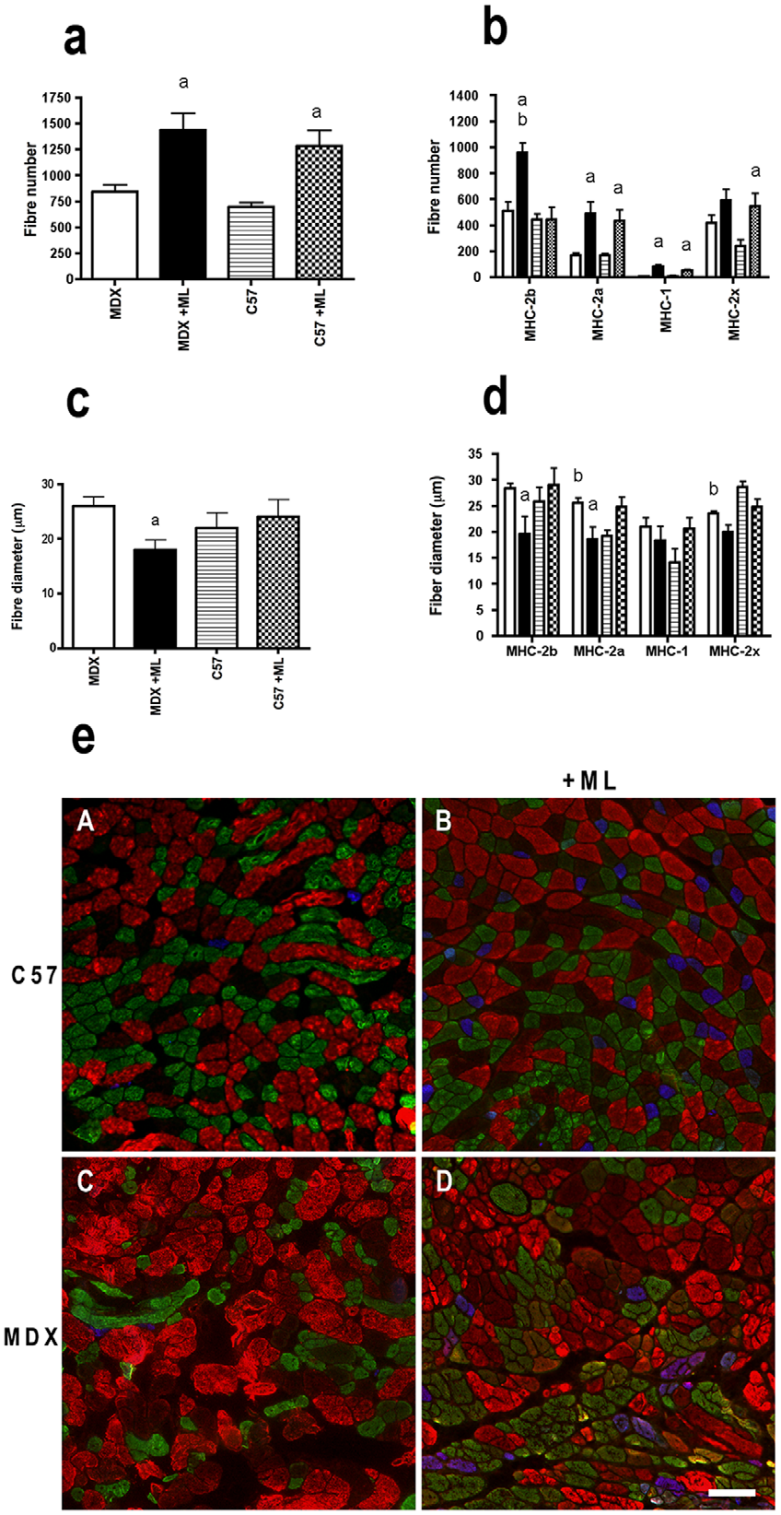
Cellular aspects of the attenuation of muscle hypertrophy following 2 month-ML in MDX mice. Number (a and b) and diameter (c,d and e) of the muscle fibers after 2 months of ML. Of note, since muscle fiber can express more than one MHC, the sum of the number of the different types of muscle fibers exceeded the total number of fibers. The representative images (e) show reduced size of fibers expressing MHC-2b following ML. Cross-sections were revealed for MHC-2b (red), MHC-2a (green) and MHC-1 (blue) reactivity. Scale bar = 20 µm. a: significantly different from unoverloaded muscle (p<0.05). b: significantly different from corresponding C57 mice (p<0.05). n = 5–8/group.

To further analyze the reduced muscle hypertrophy in MDX+ML mice in response to ML, we counted the fiber number observed in a muscle cross-section, using laminin labeling 2 months after ML. We found that in response to ML, the fiber number increased in both MDX+ML mice and C57 +ML mice, with no notable difference between the two mice strains (+71% versus + 84%)(Figure 3a). In contrast to C57+ML mice, MDX+ML mice increased the number of fibers expressing MHC-2b and did not change those expressing MHC-2x (Figure 3b). In both strains however, the number of fibers expressing MHC-2a or MHC-1 increased (Figure 3b).

Moreover, we examined the size (diameter) of the fibers 2 months after ML to confirm that the reduced muscle hypertrophy in MDX+ML mice resulted from a smaller fiber diameter in MDX+ML mice. This was in fact the case since the mean diameter of fibers in MDX+ML mice was reduced by ML (Figure 3c). However, this was not explained by a smaller percentage of large diameter fibers expressing MHC-2b (Figure 3d), but by the diameter of the fibers expressing MHC-2b or MHC-2a that was reduced by ML in MDX+ML mice (Figures 3d–e).

Together with the anabolic processes (regulated by the mTOR pathway, see below), the catabolic processes control muscle size. Thus, we addressed the role of catabolic processes in the reduced muscle hypertrophy (reduced diameter of the fibers) induced by ML in MDX mice. We studied the ubiquitin-proteasomal pathway responsible for the myofibrillar muscle protein degradation, possibly more activated in MDX+ML as compared to C57+ML. This pathway is negatively controlled via a set of E3 ligase-encoding genes, such as atrogin-1 [55]. Atrogin-1 mRNA decreased globally less in response to ML in MDX+ML than in C57+ML mice (Figure S2a). Autophagy, involving a battery of genes such as LC3 and Bnip3, also plays an important role in the degradation of muscle proteins [55]. We found that LC3 mRNA similarly decreased in MDX+ML and C57+ML mice (at day 7 respectively –31% versus -33%)(Figure S2b). However Bnip3 mRNA decreased to a somewhat lesser extent in MDX+ML as compared to C57+ML (−63% versus −82% at day 7)(Figure S2c).

### Altered anabolic signaling pathways

**Figure 4:**
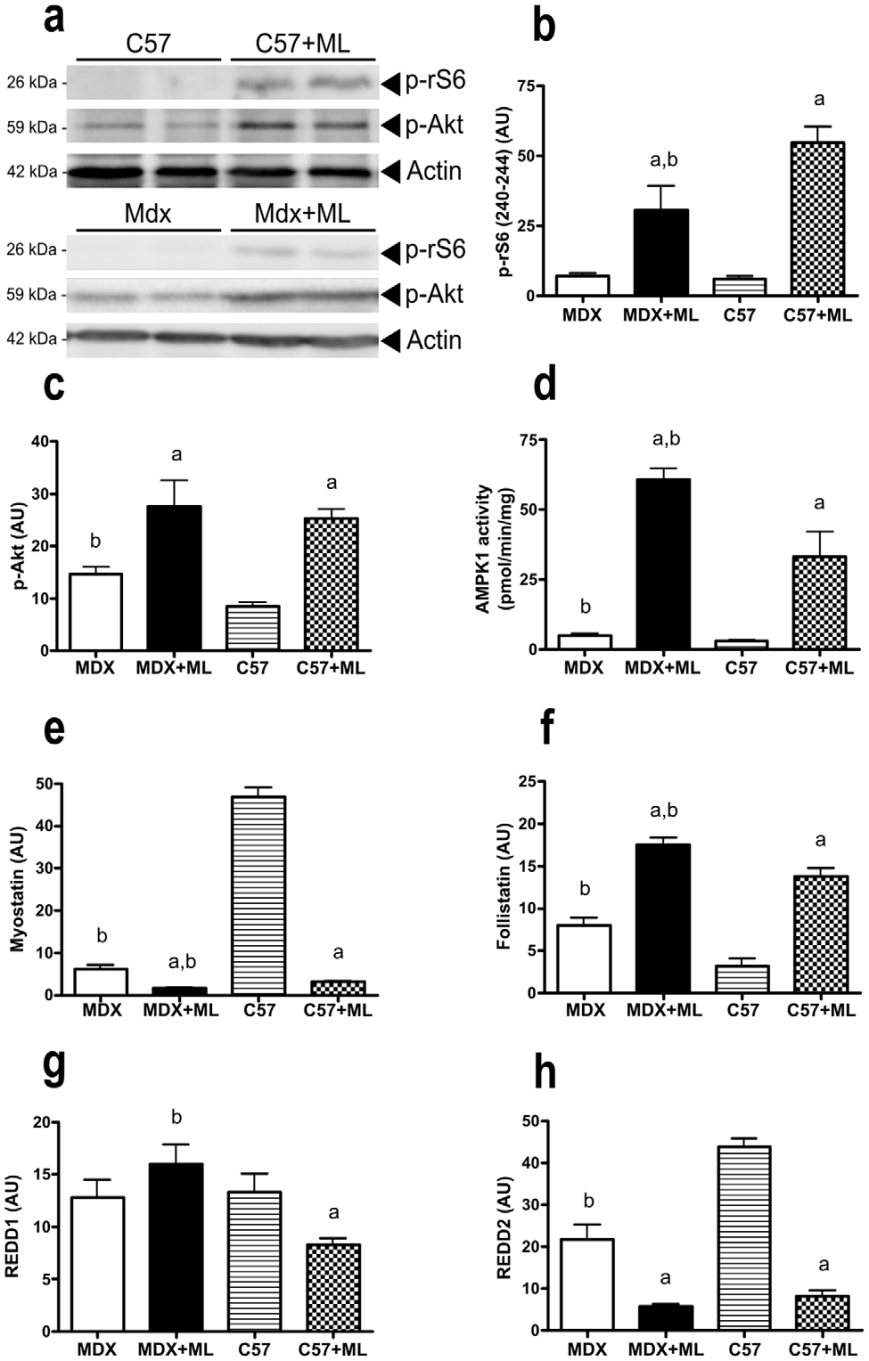
Altered activation (p-rS6 and p-Akt) and regulation (AMPKα1, myostatin, follistatin, REDD1 and REDD2) of mTOR signaling pathway at 7 days following ML in MDX mice. p-rS6 (a, b), p-Akt (a, c), AMPKα1 activity (d), myostatin (e), follistatin (f), REDD1 (g) and REDD2 (h) mRNA after 7 days of ML**.** The image (a) show representative blots of p-rS6 and p-Akt. a: significantly different from unoverloaded muscle (p<0.05). b: significantly different from corresponding C57 mice (p<0.05). n = 5–12/group.

To determine whether the reduced hypertrophy in response to ML in MDX+ML mice is also caused by a lower activation of mTOR signaling, we measured the phosphorylation level of the key downstream effector of mTOR, rS6, after 7 days of ML [10], [41], [56]. We found a smaller increase in rS6 phosphorylation in MDX+ML mice (+360%) as compared to C57+ML mice (+796%)(Figures 4a–b). Of note the level of rS6 phosphorylation was lower in MDX+ML mice than in C57+ML mice. In line with this finding, we also observed that ML caused Akt phosphorylation to a reduced extent in MDX+ML mice (+88%) than in C57+ML mice (+198%)(Figure 4c).

We measured the AMPKα1 activity at day 7 [10], [41] to test the hypothesis that the reduced mTOR activation was due to hyper-activation of AMPKα1 activity. We found that AMPKα1 activity was increased in a similar way in MDX+ML mice (+1,141%) and C57+ML mice (+1,006%) 7 days after ML; however AMPKα1 activity was higher in MDX+ML mice as compared to C57+ML mice (Figure 4d).

We also determined whether there was a smaller reduction in myostatin expression, a mTOR deactivator, in MDX+ML mice in response to ML as compared to C57+ML mice at day 7. We observed that the level of myostatin mRNA decreased less in the MDX+ML mice in response to ML (−74%) than in the C57+ML mice (−93%)(Figure 4e). Follistatin is known to antagonize myostatin, to be regulated by mTOR and to play an important role as a myoblast fusion/growth promoting factor [15]. We found that the increase in follistatin mRNA induced by 7-day ML was reduced in MDX+ML (+119%) as compared to C57+ML (+331%)(Figure 4f).

Both REDD1 and REDD2 genes (regulated in development and DNA damage responses 1 and 2) are negative regulators of mTOR signaling, REDD2 overexpression diminishing the activation of mTOR in response to mechanical signal [16]. Therefore, we also measured the expression of both genes at day 7. In contrast to C57+ML mice, there was no ML-induced decrease in REDD1 mRNA in MDX+ML mice (Figure 4g). Concerning REDD2 mRNA, it was reduced in a similar way in MDX+ML mice (−73%) and C57+ML mice (–82%) 7 days after ML (Figure 4h).

### Dystrophin rescue restored the adaptive response

Together, our results suggest that dystrophin is essential for a normal response to ML. To further substantiate the role of dystrophin in the adaptive response to ML, we performed a dystrophin rescue experiment. We evaluated the effect of U7-mediated dystrophin (U7-DYS) rescue in plantaris muscle [44]. We injected U7-DYS contruct to MDX mice 1 month before ML (MDX+ML+DYS) and we studied the muscle following 1-month ML.

**Figure 5:**
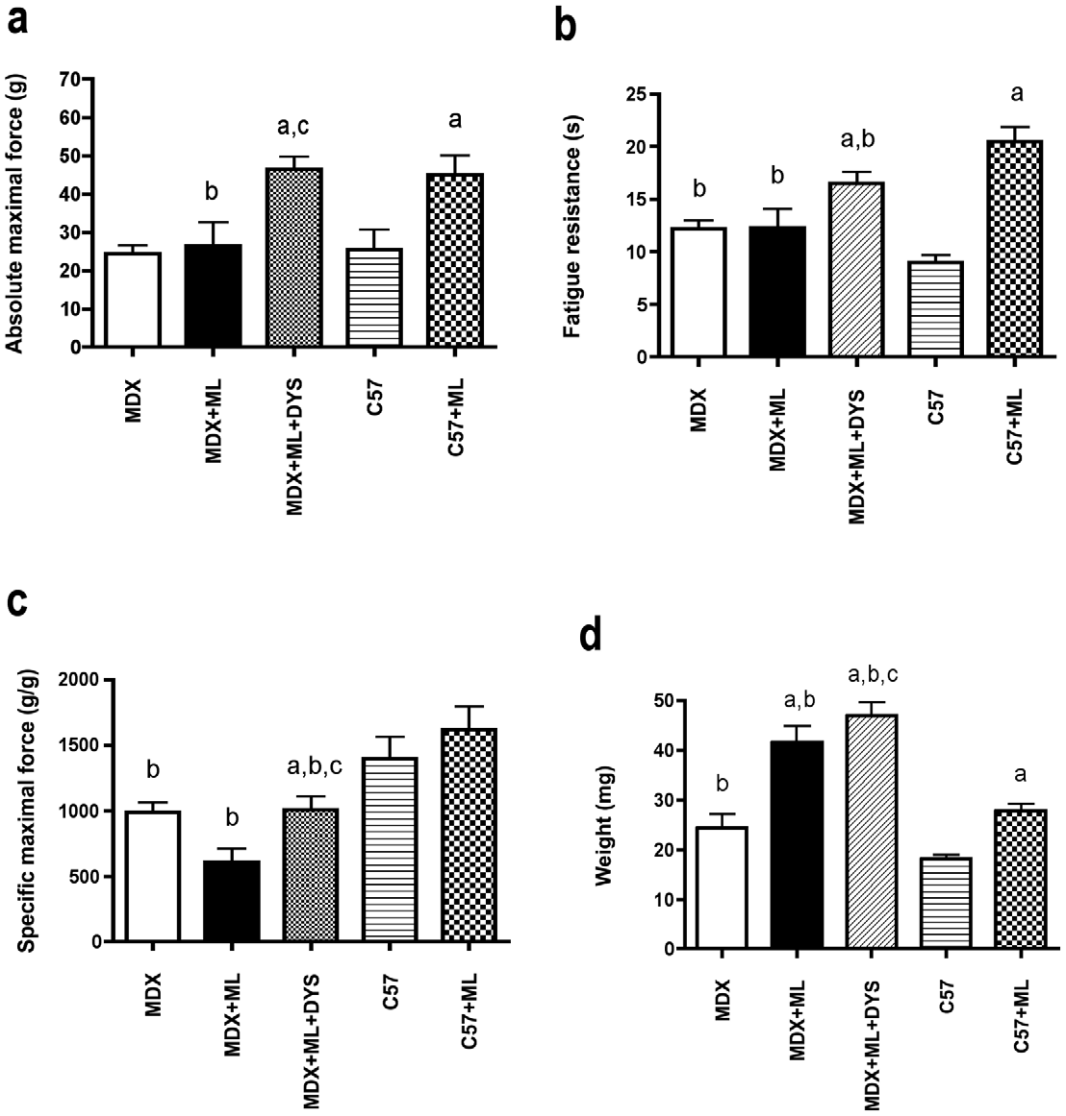
Restoration of the adaptive response to 1 month ML by dystrophin rescue. Absolute (a) and specific (b) muscle maximal forces, weight (c) and fatigue resistance (d) and after 1-month of ML combined with U7-mediated dystrophin rescue. a: significantly different from unoverloaded muscle (p<0.05). b: significantly different from corresponding C57 mice (p<0.05). c: significantly different from MDX+ML (p<0.05). n = 6/group.

In contrast to C57+ML mice, MDX+ML mice did not increase absolute maximal force and fatigue resistance and reduce specific maximal force in response to 1-month ML (Figures 5a–c), confirming that the adaptative response was blunted in MDX+ML mice as compared to C57+ML mice. MDX+ML+DYS muscles immunostained for dystrophin expression revealed high levels of dystrophin expression properly localized to the fiber sarcolemma (Figure S3a), confirming that dystrophin rescue was effective. Interestingly, the delivery of U7-DYS construct in MDX mice was able to fully restore the gain of absolute maximal force induced by 1 month ML. Indeed, ML combined with U7-DYS in MDX induced similar increase in absolute maximal force (+90%) as ML alone in C57 mice (+77%). This was explained by the fact that U7-DYS prevented the drop in specific maximal force in MDX+ML+DYS (Figure 5c), without affecting fibrosis (Figure S3b–c). Moreover, U7-DYS partially restored the increase in muscle fatigue resistance induced by ML in MDX+ML+DYS mice (Figure 5b). Finally, it induced a modest increase in muscle weight in MDX+ML+DYS (Figure 5d) that was associated with a greater diameter for the fibers expressing dystrophin as compared to the fibers not expressing dystrophin (Figure S3d).

## Discussion

In the present study, we reported for the first time that the muscle adaptive response following ML was impaired in MDX mice and dystrophin rescue restored it. Our study strongly suggests that dystrophin plays an important role in the muscle performance gain and remodeling following ML.

### Reduced performance gain

Muscle performance was improved to a lesser extent in MDX+ML mice as compared to C57+ML mice. The markedly reduced increase in muscle absolute maximal force (both 1 and 2 months after ML; at month 2:+40% versus+204%) resulted from the fact that muscle specific maximal force was reduced (1 month-ML) or did not increase (2 month-ML) in MDX mice. The reduced specific maximal force of the fast fibers in MDX+ML mice explains this difference between the two mice strains. We would suggest that the reduced specific maximal force of the muscle fibers was caused by a lower concentration of contractile proteins since we found that fibers were atrophied in the MDX+ML mice and dystrophin rescue increased their diameter (see below). In addition, we cannot exclude the possibility that ML increased the alterations in subcellular sarcomere microarchitecture in MDX mice [57]. The smaller increase in muscle absolute maximal force induced by 2 month-ML in MDX+ML mice was also caused by a reduced muscle hypertrophy (see below).

Together with the reduced gain in muscle absolute maximal force, we also reported an attenuated increase in muscle fatigue resistance in response to ML in MDX mice (both at 1 and 2 months; at 2 months:+36 versus +85%). This was associated with a reduction in the fast/glycolytic to slow/oxidative fiber-type conversion in response to ML (higher percentage of fibers expressing MHC-2b) in MDX mice. It is well established that slow/oxidative fiber are more fatigue resistant than fast/glycolytic fibers [20]. Since the calcineurin, PGC-1α, and PPAR signaling pathways promote the formation of slow/oxidative/fatigue resistant fiber [3], these pathways could be less activated in response to ML in MDX mice. The data concerning the expression of NFATc, PGC-1α and PPAR-ß mRNAs in response to ML in both strains did not support this hypothesis. However, activation of these signaling pathways, in addition to modulation of gene transcripts as analyzed in the present study, involves also post-transcriptional modifications.

Interestingly, dystrophin rescue restored these aspects of the adaptive response to ML, substantiating the idea that the absence of dystrophin is responsible of the defect. It should be noted that partial dystrophin replacement appears to be enough, confirming previous studies [58]. Apparently, the dystrophic state does not play a role in the reduced adaptive response since dystrophin rescue restores it, whithout affecting the level of fibrosis. Therefore, a key finding of the present study is that dystrophin is very likely a new player in the adaptive response to ML.

### Potential mechanisms of the fiber atrophy

In the present study, the reduced muscle hypertrophy (increase in muscle weight) in response to 2 month-ML in MDX mice is not caused by a smaller increase in fiber number per muscle cross-sectional area (similar increase). Fiber number increase following ML have been previously reported [7], [19], [24], [40], and most probably results from both de novo fiber formation from satellite cells and fiber splitting/branching since the increased fiber number is only partially attenuated with satellite cell ablation [19]. This is the atrophy of the fibers expressing MHC-2b and MHC-2a that is responsible for the reduced muscle hypertrophy in MDX+ML mice. This may result from the lower reduction in catabolic processes (ubiquitin-proteasomal pathway and autophagy) in MDX+ML, in agreement with the lower increase in Akt phosphorylation. It is also likely that split/branched and newly formed fibers did not grow enough in the MDX+ML mice. A possible lower contribution of satellite cells to muscle hypertrophy resulting from dystrophic environment is not supported by our results concerning the increase in fiber number (similar between the 2 strains) and the effect of dystrophin rescue (restoration of the adaptive response without notable change in the dystrophic environment), and the recent finding that satellite cells are not necessary for robust ML-induced hypertrophy [19].

The increase in fiber number following ML is independent of mTOR signaling whereas fiber growth is not [6]. Therefore, we propose that the splitted/branched and newly formed fibers did not grow enough in the MDX+ML mice due to reduced mTOR signaling in response to ML. In support of this hypothesis, we found a lower increase in phosphorylation of rS6 in MDX+ML mice. Moreover, phosphorylation of Akt is also increased to a lesser extent; activation of Akt also promoting muscle hypertrophy [8], [9]. The reduced augmentation of follistatin and the reduced attenuation of myostatin and REDD1 in response to ML in MDX mice were consistent with the inhibition of mTOR activation [12], [13], [15], [16]. We have recently reported that AMPKα1 plays a key role in suppressing mTOR activation and limits hypertrophy in response to ML [10]. In the present study, we found that AMPKα1 activity increased to a similar extent in response to ML in MDX mice as compared to C57+ML; however AMPKα1 actvity was higher in MDX+ML mice.

Together with an inhibition of the mTOR pathway, it is also possible that reduced mTOR signaling in MDX+ML mice resulted from a lower input from mTOR activation during ML. Recent studies have ruled out the theory that local production of insulin-like growth factor (IGF-1) and IGF-1 signaling is responsible for mTOR activation in response to ML [17], [43], [59]. This is consistent with the fact that IGF-1 does not either cause hypertrophy nor activate Akt/mTOR signaling in (adult) muscle [60]. How mTOR signaling is activated by mechanical signals is not well understood [2], [61]. However, mTOR activation in response to ML is reduced by inhibition of mechanoreceptors such as stretch-activated channels [62]. Therefore, the lower activation of mTOR in MDX+ML mice may result from a dysfunction in the mechanoreceptors due to colocalization of dystrophin and mechanoreceptors [30], [31].

Since dystrophin rescue increased the fiber diameter in MDX+ML+DYS mice, this result substantiates the idea that dystrophin plays an important role both in anabolic and catabolic pathways, possibly due to the fact that dystrophin contributes to force transmission [26] and serves as a scaffold for signaling complexes [29], [30], [31]. Consistent with our results, it was reported that dystrophin may be critical for muscle growth/maintenance [63], [64].

### Conclusion

The extent of the improvement in muscle performance (gains in absolute maximal force and fatigue resistance) in response to ML was lower in dystrophic MDX mice. Decreased cellular and molecular adaptations (hypertrophy, promotion of slower/oxidative fibers) in response to ML and altered intracellular signaling pathways involved in muscle growth and maintenance were also found in MDX mice. The fact that dystrophin rescue restored the muscle response to ML further substantiates the idea that dystrophin plays an important role. Future studies are required to dissect the detailed link between dystrophin and muscle growth and maintenance. Additional studies are also needed to test the possibility that long-term physical rehabilitation using high-force contractions could be a safe therapeutic strategy to ameliorate dystrophic muscle weakness.

## Methods

### Animals and mechanical overloading

All procedures were performed in accordance with national and European legislations. Male MDX mice (MDX, C57BL/10ScSc-DMD^mdx^/J) and their control (C57, C57BL/10) were used at 4–6 months of age. The *MDX* mice were kindly provided by D. Sassoon and A. Pannerec (INSERM U787 Paris). All animals were housed in conventional conditions. The animals were anaesthetized with pentobarbital (ip, 50 mg/kg body weight). The plantaris muscles of both legs from each mouse were mechanically overloaded (ML) by the surgical removal of soleus muscles and a major portion of the gastrocnemius muscles as described [39]. Body weight was not changed by 2 month ML in both MDX and C57 mice. The plantaris muscles were recovered 3 and 7 days, and 1 and 2 months following ML. The animals were euthanized with an overdose of pentobarbital.

### Dystrophin rescue strategy

The restoration of a quasi-dystrophin was mediated by the vectorized U7 exon-skipping technique (U7-DYS)[44]. Vectors were prepared according to published protocols [45]. Adeno-associated vectors (AAV2/1) carrying the U7-DYS construct were injected in 6 MDX animals through intra-arterial perfusion of the right hind limb. Titer for AAV2/1-U7 was 1.10^12^ vector genomes (vg).ml^-1^. Detailed procedure for intra-arterial injection was previously described [46]. Briefly, anesthetized mice (2–4% isoflurane) underwent femoral artery and vein isolation of the right hindlimb. After clamping the femoral vein and two collaterals, a catheter was introduced in the femoral artery and we injected 1 ml per 20 g of body weight at a rate of 100 µl.s^-1^. Control muscle was obtained from left hind limb in which femoral artery was injected with saline solution only. Muscles were collected 1 month after ML.

### Whole muscle force measurement

Skeletal muscle function was evaluated by measuring *in situ* muscle contraction, as described previously [47], [48]. At different times after ML (1 and 2 months after ML), animals were anesthetized (ip, pentobarbital sodium, 50 mg/kg). During physiological experiments, supplemental doses were given as required to maintain deep anesthesia. The knee and foot were fixed with clamps and stainless steel pins. The plantaris muscle was exposed and the distal tendon of the gastronecmius and soleus muscle complex was cut. The distal tendon of the plantaris muscle was attached to an isometric transducer (Harvard Bioscience) using a silk ligature. The sciatic nerves were proximally crushed and distally stimulated by a bipolar silver electrode using supramaximal square wave pulses of 0.1 ms duration. Responses to tetanic stimulation (pulse frequency 50–143 Hz) were successively recorded. At least 1 min was allowed between contractions. Absolute maximal forces were determined at optimal length (length at which maximal tension was obtained during the tetanus). Force was normalized to the muscle mass (m) as an estimate of specific maximal force. Fatigue resistance was then determined after a 5-min rest period. The muscle was continuously stimulated at 50 Hz for 2 min (sub maximal continuous tetanus). The duration corresponding to a 20% decrease in force was noted. Body temperature was maintained at 37°C using radiant heat.

### Single muscle fiber force measurement

Plantaris muscles were collected 2 months after ML and immediately placed in an ice-cold relax solution (in mmol/l: 100 KCl, 20 Imidazole, 7 MgCl_2_, 2 EGTA, 4 ATP, pH 7.0; 4°C). Small bundles of ∼25–50 fibers were dissected free from the muscle and tied to a glass micro capillary tube at ∼110% resting length. The bundles were then placed in a skinning solution (relax solution containing glycerol; 50∶50 v/v) at 4°C for 24 h and subsequently treated with a cryoprotectant (sucrose solution) for long-term storage at −80°C as described earlier [49]. On the day of experiment, a bundle was desucrosed and single fibers isolated. A fiber segment length of 1 to 2 mm was then left exposed to the relax solution between connectors leading to a force transducer (model 400A, Aurora Scientific) and a lever arm system (model 308B, Aurora Scientific). The apparatus was mounted on the stage of an inverted microscope (model IX70; Olympus). While the fiber segment was in relax solution, sarcomere length was set to 2.50±0.05 µm by adjusting the overall segment length. The sarcomere length was controlled during the experiments using a high-speed video analysis system (model 901A HVSL, Aurora Scientific). The fiber segment width, depth and length between the connectors were measured. Fiber cross-sectional area (CSA) was calculated from the diameter and depth, assuming an elliptical circumference, and was corrected for the 20% swelling that is known to occur during skinning. At 15°C, immediately preceding each activation, the fiber segment was immersed for 10–20 s in a solution with a reduced Ca^2+^-EGTA buffering capacity. This solution is identical to the relax solution except that the EGTA concentration is reduced to 0.5 mM, which results in more rapid attainment of steady force during subsequent activation. Maximal isometric force was calculated as the difference between the total force in activating solution (pCa 4.5) and the resting force measured in the same segment while in the relax solution. Maximal force was adjusted for fiber CSA (specific maximal force). After mechanical measurements, each fiber was placed in urea buffer in a plastic microcentrifuge tube and stored at −80°C until analyzed by gel electrophoresis. The myosin heavy chain (MHC) isoform composition of fibers was determined by 6% SDS-PAGE. The acrylamide concentration was 4% (wt/vol) in the stacking gel and 6% in the running gel, and the gel matrix included 30% glycerol. Sample loads were kept small (equivalent to ∼0.05 mm of fiber segment) to improve the resolution of the MHC bands (slow and fast MyHC: type I, IIa, IIx and IIb). Electrophoresis was performed at 120 V for 24 h with a Tris–glycine electrode buffer (pH 8.3) at 15°C (SE 600 vertical slab gel unit, Hoefer Scientific Instruments). The gels were silver-stained and subsequently scanned in a soft laser densitometer (Molecular Dynamics) with a high spatial resolution (50 µm pixel spacing) and 4096 optical density levels.

### Histology

Transverse serial sections (8 µm) of muscles (collected 1 and 2 months after ML) were obtained using a cryostat, in the mid-belly region. The muscles were sectioned at different intervals to determine the maximal muscle CSA and the corresponding sections were studied. Some of the sections were processed for histological analysis according to standard protocols (stained for H&E, Sirius red, dapi). Others were used for immunohistochemistry as described previously [50], [51]. For determination of muscle fiber diameter and myosin heavy chain (MHC) analysis, frozen unfixed sections were blocked 1 h in PBS plus 2% BSA, 2% sheep serum and incubated 30 min with mouse Fab 1/100 in PBS. Sections were then incubated overnight with primary antibodies against laminin (Z0097, Dako), dystrophin (anti-Dys1, Novocastra) and MHC isoforms (BAD5/MHC-1, BF3/MHC-2b, SC71/MHC-2a, 6H1/MHC-2x, F1652/MHC-dev, Hybridoma bank). After washes in PBS, sections were incubated 1 h with secondary antibodies (alexa fluor). After washes in PBS, slides were finally mounted in Fluoromont (Southern Biotech). Morphometric analyses were made on two serial sections of muscles. Images were captured using a digital camera (Hamamatsu ORCA-AG) attached to a motorized fluorescence microscope (Zeiss AxioImager.Z1), and morphometric analyses were made using the software MetaMorph 7.5 (Molecular Devices). The smallest diameter of the fibers was measured. For muscle fiber diameter and fiber typing analyses all of the muscle fibers of the muscle section were measured. Unfortunately, it was not possible to distinguish pure and hybrid fibers expressing MHC-2b and MHC-2x since the isotype and species of the 2 primary antibodies were similar. To evaluate the amount of fibrosis, we measured the cross-sectional area occupied by Sirius red stained interstitial tissue.

### AMPK activity assay

Muscles were recovered 7 days after ML (they were not previously electrically stimulated) in order to assay AMK activity. AMPK was immunoprecipitated from 30 µg lysate with antibodies against the α1 catalytic subunit and assayed for phosphotransferase activity towards *AMARA* peptide (AMARAASAAALARRR) using [γ-^32^P]-ATP as previously described [52]. AMPKα1 antibodies used for immunoprecipitation was generated and donated by Professor D. Grahame Hardie (University of Dundee)

### Western blot analysis

Immunoblotting was carried out as described previously [47], [53] using muscles 7 days after ML (muscles were not previously electrically stimulated) from overnight fasted mice. Muscle tissues were snap frozen in liquid nitrogen immediately after dissection. Frozen muscles were placed into an ice-cold homogenization buffer containing: 50 mM Tris (pH 7.6), 250 mM NaCl, 3 mM EDTA, 3 mM EGTA, 0.5% NP40, 2 mM dithiothreitol, 10 mM sodium orthovanadate, 10 mM NaF, 10 mM glycerophosphate and 2% of protease inhibitor cocktail (Sigma, P8340). Samples were finely minced with scissors and then homogenized using plastic pestles, incubated 30 minutes on ice, sonicated 3 times for 5 s with 30 s intervals on ice and then centrifuged at 12,000 *g* for 30 min at 4°C. Protein concentration was measured using the Bradford method with BSA as a standard. Equal amounts of protein extracts, ie. 10 µg (phosphorylated-Ak, p-Akt), or 20 µg (phosphorylated-ribosomal S6 protein, p-rS6) were separated by SDS-PAGE before electrophoretic transfer onto a nitrocellulose membrane (Amersham Hybond-ECL, GE Healthcare). Western-blot analysis was carried out using p-rS6 (Ser240/244) antibody (1∶2000, Cell Signaling #2215S); p-Akt (Ser473) antibody (1∶2000, Cell Signaling #9271S), and a pan-actin Antibody (Clone C4, 1∶20000, Millipore). Antibody reacting bands were visualized with peroxidase-conjugated secondary antibodies (Pierce Biotechnology) and a chemiluminescent detection system (ECL-Plus; GE Healthcare). Bands of actin were used to check that the protein load was correct. Bands were quantified by densitometric software (Multi Gauge, Fujifilm).

### Relative quantification of gene expression by qPCR

Unstimulated muscles were collected 3 and 7 days after ML. Total RNA was extracted from plantaris muscle using miRNeasy Mini Kit (Qiagen SA) following the manufacturer's instructions. RNA quality for each sample was checked with Experion RNA StdSens Analysis Kit and the Experion automated electrophoresis station according to the manufacturer's instructions. Only samples with a RQI (RNA quality indicator) superior to 7 were used for further studies. The first-strand cDNA was then synthesized using Transcriptor First Strand cDNA Synthesis Kit (Roche Diagnostics) with Anchored-oligo(dT)_18_ primer and according to the manufacturer's instructions. PCR analysis was then carried out with SYBR green PCR technology using Light Cycler 480 system (Roche Diagnostics). The reaction was carried out in duplicate for each sample in a 8 µl reaction volume containing 4 µl of SYBR Green Master Mix and 500 nM each the forward and reverse primer, and 4 µl of diluted (1∶25) cDNA. The thermal profile for SYBR Green qPCR was 95°C for 8 min, followed by 40 cycles at 95°C for 15 s, 60°C for 15 s, 72°C 30 s. Primers sequences used in this study are available on request. The gene expression stability of GAPDH, HPRT and P0 were calculated using geNorm software and after analysis only HPRT and P0 were used as the references transcripts.

### Statistical analysis

Groups were statistically compared using analysis of variance. If necessary, subsequent contrast analysis was also performed. For groups that did not pass tests of normality and equal variance, non-parametric tests were used (Kruskal Wallis and Wilcoxon). Values are means ± SEM.

## Supporting Information

Figure S1 **Factors promoting fast/glycolytic to slow/oxidative fiber-type conversion after 7 days of ML.** PPARß (a), PGC1α (b) and NFATc1a (c) mRNA after 7 days of ML. a: significantly different from unoverloaded muscle (p<0.05). b: significantly different from corresponding C57 mice (p<0.05). n = 6/group.
(TIF)

Figure S2 **Changes in catabolic processes following ML in MDX mice after 3 and 7 days of ML.** Atrogin-1 (ubiquitin-proteasomal pathway)(a), LC3 (b) and Bnip3 (autophagy)(c) mRNA at 3 and 7 days. a: significantly different from unoverloaded muscle (p<0.05). b: significantly different from corresponding C57 mice (p<0.05). n = 5–10/group.
(TIF)

Figure S3 **Cellular effects of dystrophin rescue at 1 month following ML in MDX mice.** (a) representative image of fibers expressing dystrophin in MDX+ML+DYS mice, (b, c) fibrosis using red Sirius staining and (d) diameter of fibers expressing (DYS positive) or not dystrophin (DYS negative) in MDX+ML mice. c: significantly different from fiber expressing not dystrophin (p<0.05). n = 4–6/group.
(TIF)

## References

[1] Miyazaki M, Esser KA (2009). Cellular mechanisms regulating protein synthesis and skeletal muscle hypertrophy in animals.. J Appl Physiol.

[2] Spangenburg EE (2009). Changes in muscle mass with mechanical load: possible cellular mechanisms.. Appl Physiol Nutr Metab.

[3] Schiaffino S, Sandri M, Murgia M (2007). Activity-dependent signaling pathways controlling muscle diversity and plasticity.. Physiology (Bethesda).

[4] Fluck M, Hoppeler H (2003). Molecular basis of skeletal muscle plasticity–from gene to form and function.. Rev Physiol Biochem Pharmacol.

[5] Gundersen K (2010). Excitation-transcription coupling in skeletal muscle: the molecular pathways of exercise.. Biol Rev Camb Philos Soc.

[6] Bodine SC, Stitt TN, Gonzalez M, Kline WO, Stover GL (2001). Akt/mTOR pathway is a crucial regulator of skeletal muscle hypertrophy and can prevent muscle atrophy in vivo.. Nat Cell Biol.

[7] Goodman CA, Frey JW, Mabrey DM, Jacobs BL, Lincoln HC (2011). The role of skeletal muscle mTOR in the regulation of mechanical load-induced growth.. J Physiol.

[8] Lai KM, Gonzalez M, Poueymirou WT, Kline WO, Na E (2004). Conditional activation of akt in adult skeletal muscle induces rapid hypertrophy.. Mol Cell Biol.

[9] Blaauw B, Canato M, Agatea L, Toniolo L, Mammucari C (2009). Inducible activation of Akt increases skeletal muscle mass and force without satellite cell activation.. Faseb J.

[10] Mounier R, Lantier L, Leclerc J, Sotiropoulos A, Pende M (2009). Important role for AMPKalpha1 in limiting skeletal muscle cell hypertrophy.. Faseb J.

[11] Xu Z, Ichikawa N, Kosaki K, Yamada Y, Sasaki T (2010). Perlecan deficiency causes muscle hypertrophy, a decrease in myostatin expression, and changes in muscle fiber composition.. Matrix Biol.

[12] Trendelenburg AU, Meyer A, Rohner D, Boyle J, Hatakeyama S (2009). Myostatin reduces Akt/TORC1/p70S6K signaling, inhibiting myoblast differentiation and myotube size.. Am J Physiol Cell Physiol.

[13] Sartori R, Milan G, Patron M, Mammucari C, Blaauw B (2009). Smad2 and 3 transcription factors control muscle mass in adulthood.. Am J Physiol Cell Physiol.

[14] Amirouche A, Durieux AC, Banzet S, Koulmann N, Bonnefoy R (2009). Down-regulation of Akt/mammalian target of rapamycin signaling pathway in response to myostatin overexpression in skeletal muscle.. Endocrinology.

[15] Sun Y, Ge Y, Drnevich J, Zhao Y, Band M (2010). Mammalian target of rapamycin regulates miRNA-1 and follistatin in skeletal myogenesis.. J Cell Biol.

[16] Miyazaki M, Esser KA (2009). REDD2 is enriched in skeletal muscle and inhibits mTOR signaling in response to leucine and stretch.. Am J Physiol Cell Physiol.

[17] Miyazaki M, McCarthy JJ, Fedele MJ, Esser KA (2011). Early activation of mTORC1 signalling in response to mechanical overload is independent of phosphoinositide 3-kinase/Akt signalling.. J Physiol.

[18] Dupont E, Cieniewski-Bernard C, Bastide B, Stevens L (2011). Electrostimulation during hindlimb unloading modulates PI3K-AKT downstream targets without preventing soleus atrophy and restores slow phenotype through ERK.. Am J Physiol Regul Integr Comp Physiol.

[19] McCarthy JJ, Mula J, Miyazaki M, Erfani R, Garrison K (2011). Effective fiber hypertrophy in satellite cell-depleted skeletal muscle.. Development.

[20] Burke RE, Levine DN, Tsairis P, Zajac FE (1973). Physiological types and histochemical profiles in motor units of the cat gastrocnemius.. J Physiol.

[21] Bassel-Duby R, Olson EN (2006). Signaling pathways in skeletal muscle remodeling.. Annu Rev Biochem.

[22] Dunn SE, Burns JL, Michel RN (1999). Calcineurin is required for skeletal muscle hypertrophy.. J Biol Chem.

[23] Dunn SE, Chin ER, Michel RN (2000). Matching of calcineurin activity to upstream effectors is critical for skeletal muscle fiber growth.. J Cell Biol.

[24] Parsons SA, Millay DP, Wilkins BJ, Bueno OF, Tsika GL (2004). Genetic loss of calcineurin blocks mechanical overload-induced skeletal muscle fiber type switching but not hypertrophy.. J Biol Chem.

[25] Lira VA, Benton CR, Yan Z, Bonen A (2010). PGC-1alpha regulation by exercise training and its influences on muscle function and insulin sensitivity.. Am J Physiol Endocrinol Metab.

[26] Ramaswamy KS, Palmer ML, van der Meulen JH, Renoux A, Kostrominova TY (2011). Lateral transmission of force is impaired in skeletal muscles of dystrophic mice and very old rats..

[27] Lynch GS, Hinkle RT, Chamberlain JS, Brooks SV, Faulkner JA (2001). Force and power output of fast and slow skeletal muscles from mdx mice 6–28 months old.. J Physiol.

[28] Dumonceaux J, Marie S, Beley C, Trollet C, Vignaud A (2010). Combination of myostatin pathway interference and dystrophin rescue enhances tetanic and specific force in dystrophic mdx mice.. Mol Ther.

[29] Friedrich O, von Wegner F, Chamberlain JS, Fink RH, Rohrbach P (2008). L-type Ca2+ channel function is linked to dystrophin expression in mammalian muscle.. PLoS One.

[30] Allen DG, Zhang BT, Whitehead NP (2010). Stretch-Induced Membrane Damage in Muscle: Comparison of Wild-Type and mdx Mice.. Adv Exp Med Biol.

[31] Rolland JF, De Luca A, Burdi R, Andreetta F, Confalonieri P (2006). Overactivity of exercise-sensitive cation channels and their impaired modulation by IGF-1 in mdx native muscle fibers: beneficial effect of pentoxifylline.. Neurobiol Dis.

[32] Sellman JE, DeRuisseau KC, Betters JL, Lira VA, Soltow QA (2006). In vivo inhibition of nitric oxide synthase impairs upregulation of contractile protein mRNA in overloaded plantaris muscle.. J Appl Physiol.

[33] Pietri-Rouxel F, Gentil C, Vassilopoulos S, Baas D, Mouisel E (2010). DHPR alpha1S subunit controls skeletal muscle mass and morphogenesis.. Embo J.

[34] Kumar A, Khandelwal N, Malya R, Reid MB, Boriek AM (2004). Loss of dystrophin causes aberrant mechanotransduction in skeletal muscle fibers.. Faseb J.

[35] Barton ER (2006). Impact of sarcoglycan complex on mechanical signal transduction in murine skeletal muscle.. Am J Physiol Cell Physiol.

[36] Stupka N, Michell BJ, Kemp BE, Lynch GS (2006). Differential calcineurin signalling activity and regeneration efficacy in diaphragm and limb muscles of dystrophic mdx mice.. Neuromuscul Disord.

[37] Nakamura A, Yoshida K, Ueda H, Takeda S, Ikeda S (2005). Up-regulation of mitogen activated protein kinases in mdx skeletal muscle following chronic treadmill exercise.. Biochim Biophys Acta.

[38] Lang JM, Esser KA, Dupont-Versteegden EE (2004). Altered activity of signaling pathways in diaphragm and tibialis anterior muscle of dystrophic mice.. Exp Biol Med (Maywood).

[39] Baldwin KM, Valdez V, Herrick RE, MacIntosh AM, Roy RR (1982). Biochemical properties of overloaded fast-twitch skeletal muscle.. J Appl Physiol.

[40] Ianuzzo CD, Gollnick PD, Armstrong RB (1976). Compensatory adaptations of skeletal muscle fiber types to a long-term functional overload.. Life Sci.

[41] McGee SL, Mustard KJ, Hardie DG, Baar K (2008). Normal hypertrophy accompanied by phosphoryation and activation of AMP-activated protein kinase alpha1 following overload in LKB1 knockout mice.. J Physiol.

[42] Roy RR, Edgerton VR (1995). Response of mouse plantaris muscle to functional overload: comparison with rat and cat.. Comp Biochem Physiol A Physiol.

[43] Spangenburg EE, Le Roith D, Ward CW, Bodine SC (2008). A functional insulin-like growth factor receptor is not necessary for load-induced skeletal muscle hypertrophy.. J Physiol.

[44] Goyenvalle A, Vulin A, Fougerousse F, Leturcq F, Kaplan JC (2004). Rescue of dystrophic muscle through U7 snRNA-mediated exon skipping.. Science.

[45] Riviere C, Danos O, Douar AM (2006). Long-term expression and repeated administration of AAV type 1, 2 and 5 vectors in skeletal muscle of immunocompetent adult mice.. Gene Ther.

[46] Gonin P, Arandel L, Van Wittenberghe L, Marais T, Perez N (2005). Femoral intra-arterial injection: a tool to deliver and assess recombinant AAV constructs in rodents whole hind limb.. J Gene Med.

[47] Mouisel E, Vignaud A, Hourde C, Butler-Browne G, Ferry A (2010). Muscle weakness and atrophy are associated with decreased regenerative capacity and changes in mTOR signaling in skeletal muscles of venerable (18–24-month-old) dystrophic mdx mice.. Muscle Nerve.

[48] Risson V, Mazelin L, Roceri M, Sanchez H, Moncollin V (2009). Muscle inactivation of mTOR causes metabolic and dystrophin defects leading to severe myopathy.. J Cell Biol.

[49] Frontera WR, Larsson L (1997). Contractile studies of single human skeletal muscle fibers: a comparison of different muscles, permeabilization procedures, and storage techniques.. Muscle Nerve.

[50] Trollet C, Anvar SY, Venema A, Hargreaves IP, Foster K (2010). Molecular and phenotypic characterization of a mouse model of oculopharyngeal muscular dystrophy reveals severe muscular atrophy restricted to fast glycolytic fibres.. Hum Mol Genet.

[51] Agbulut O, Vignaud A, Hourde C, Mouisel E, Fougerousse F (2009). Slow myosin heavy chain expression in the absence of muscle activity.. Am J Physiol Cell Physiol.

[52] Sakamoto K, Goransson O, Hardie DG, Alessi DR (2004). Activity of LKB1 and AMPK-related kinases in skeletal muscle: effects of contraction, phenformin, and AICAR.. Am J Physiol Endocrinol Metab.

[53] Hourde C, Jagerschmidt C, Clement-Lacroix P, Vignaud A, Ammann P (2009). Androgen replacement therapy improves function in male rat muscles independently of hypertrophy and activation of the Akt/mTOR pathway.. Acta Physiol (Oxf).

[54] Marino JS, Tausch BJ, Dearth CL, Manacci MV, McLoughlin TJ (2008). Beta2-integrins contribute to skeletal muscle hypertrophy in mice.. Am J Physiol Cell Physiol.

[55] Sandri M (2008). Signaling in muscle atrophy and hypertrophy.. Physiology (Bethesda).

[56] Borst SE (2004). Interventions for sarcopenia and muscle weakness in older people.. Age Ageing.

[57] Friedrich O, Both M, Weber C, Schurmann S, Teichmann MD (2010). Microarchitecture is severely compromised but motor protein function is preserved in dystrophic mdx skeletal muscle.. Biophys J.

[58] Koo T, Malerba A, Athanasopoulos T, Trollet C, Boldrin L (2011). Delivery of AAV2/9-Microdystrophin Genes Incorporating Helix 1 of the Coiled-Coil Motif in the C-Terminal Domain of Dystrophin Improves Muscle Pathology and Restores the Level of alpha1-Syntrophin and alpha-Dystrobrevin in Skeletal Muscles of mdx Mice.. Hum Gene Ther.

[59] Hamilton DL, Philp A, MacKenzie MG, Baar K (2010). A limited role for PI(3,4,5)P3 regulation in controlling skeletal muscle mass in response to resistance exercise.. PLoS One.

[60] Shavlakadze T, Chai J, Maley K, Cozens G, Grounds G (2010). A growth stimulus is needed for IGF-1 to induce skeletal muscle hypertrophy in vivo.. J Cell Sci.

[61] Hornberger TA (2011). Mechanotransduction and the regulation of mTORC1 signaling in skeletal muscle.. Int J Biochem Cell Biol.

[62] Spangenburg EE, McBride TA (2006). Inhibition of stretch-activated channels during eccentric muscle contraction attenuates p70S6K activation.. J Appl Physiol.

[63] Acharyya S, Butchbach ME, Sahenk Z, Wang H, Saji M (2005). Dystrophin glycoprotein complex dysfunction: a regulatory link between muscular dystrophy and cancer cachexia.. Cancer Cell.

[64] Judge LM, Arnett AL, Banks GB, Chamberlain JS (2011). Expression of the dystrophin isoform Dp116 preserves functional muscle mass and extends lifespan without preventing dystrophy in severely dystrophic mice.. Hum Mol Genet.

