# Correction: Impaired Adaptive Response to Mechanical Overloading in Dystrophic Skeletal Muscle

**DOI:** 10.1371/annotation/2c0f8405-a2cc-4240-8e1b-117d3ac1a154

**Published:** 2013-06-27

**Authors:** Pierre Joanne, Christophe Hourdé, Julien Ochala, Yvain Caudéran, Fadia Medja, Alban Vignaud, Etienne Mouisel, Wahiba Hadj-Said, Ludovic Arandel, Luis Garcia, Aurélie Goyenvalle, Rémi Mounier, Daria Zibroba, Kei Sakamato, Gillian Butler-Browne, Onnik Agbulut, Arnaud Ferry

The name of the 14th author was spelled incorrectly. The correct name is: Kei Sakamoto.

