# Supplementary figures and images for: Impaired Adaptive Response to Mechanical Overloading in Dystrophic Skeletal Muscle
Source: PLoS One. 2012 Apr 12;7(4):e35346. doi: 10.1371/journal.pone.0035346 (PMC3325198; doi:10.1371/journal.pone.0035346)

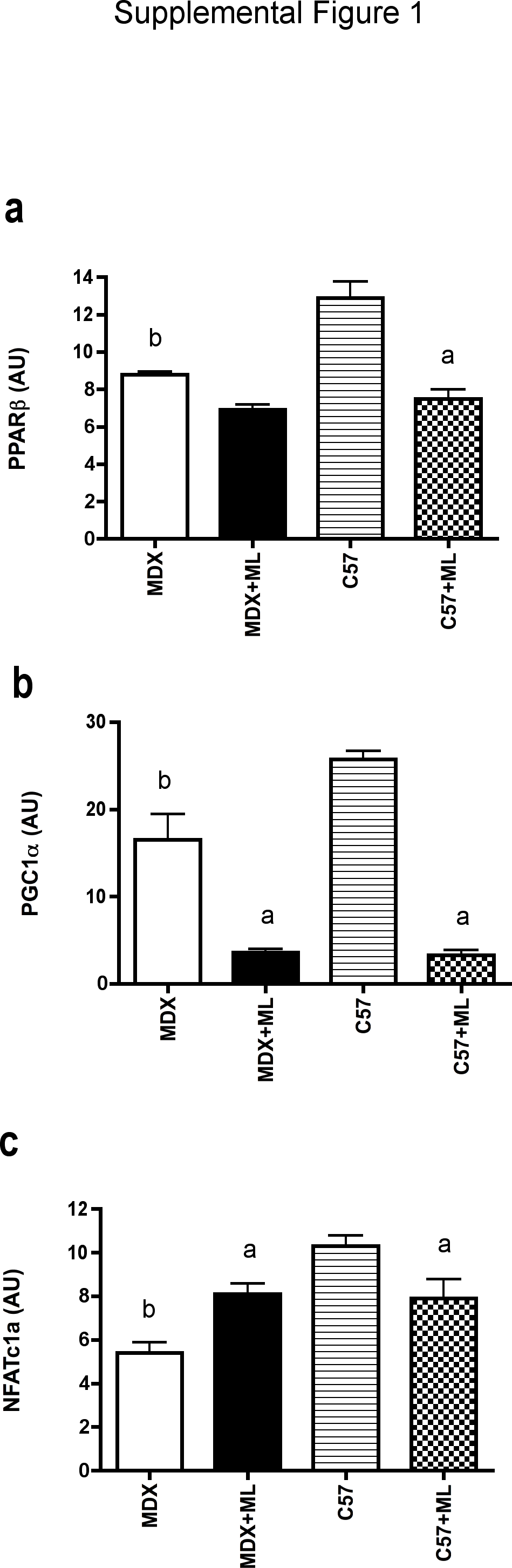

Supplement: Figure S1 — Factors promoting fast/glycolytic to slow/oxidative fiber-type conversion after 7 days of ML. PPARß (a), PGC1α (b) and NFATc1a (c) mRNA after 7 days of ML. a: significantly different from unoverloaded muscle (p<0.05). b: significantly different from corresponding C57 mice (p<0.05). n = 6/group. (TIF) [file pone.0035346.s001.tif]

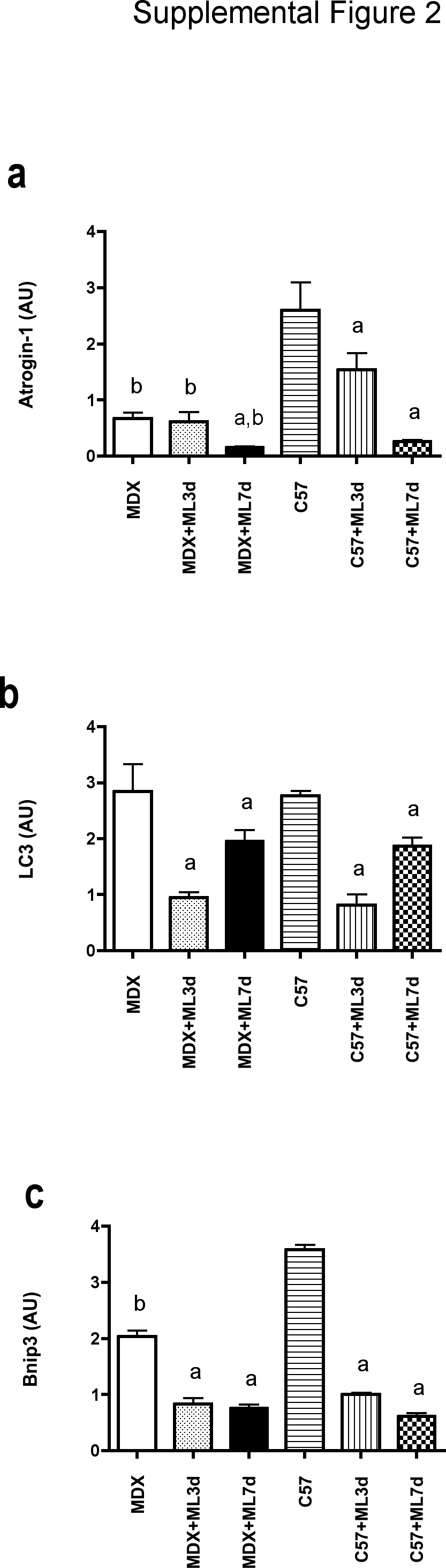

Supplement: Figure S2 — Changes in catabolic processes following ML in MDX mice after 3 and 7 days of ML. Atrogin-1 (ubiquitin-proteasomal pathway)(a), LC3 (b) and Bnip3 (autophagy)(c) mRNA at 3 and 7 days. a: significantly different from unoverloaded muscle (p<0.05). b: significantly different from corresponding C57 mice (p<0.05). n = 5–10/group. (TIF) [file pone.0035346.s002.tif]

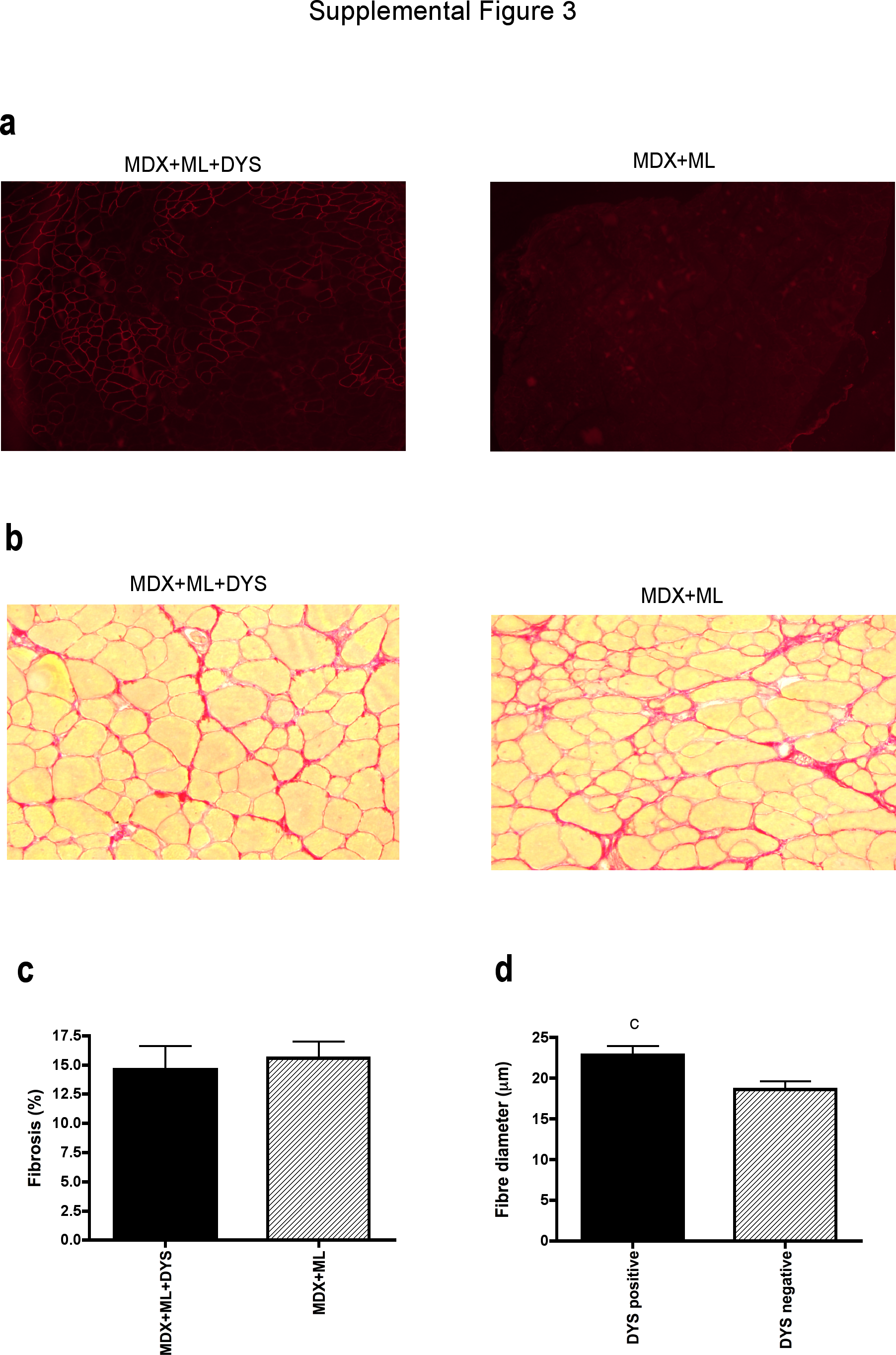

Supplement: Figure S3 — Cellular effects of dystrophin rescue at 1 month following ML in MDX mice. (a) representative image of fibers expressing dystrophin in MDX+ML+DYS mice, (b, c) fibrosis using red Sirius staining and (d) diameter of fibers expressing (DYS positive) or not dystrophin (DYS negative) in MDX+ML mice. c: significantly different from fiber expressing not dystrophin (p<0.05). n = 4–6/group. (TIF) [file pone.0035346.s003.tif]
